# Beyond the Risk of Biofilms: An Up-and-Coming Battleground of Bacterial Life and Potential Antibiofilm Agents

**DOI:** 10.3390/life13020503

**Published:** 2023-02-11

**Authors:** Mohamed Zeineldin, Ahmed Esmael, Rashad R. Al-Hindi, Mona G. Alharbi, Debebe Ashenafi Bekele, Addisu D. Teklemariam

**Affiliations:** 1 Department of Animal Medicine, College of Veterinary Medicine, Benha University, Benha 13511, Egypt; 2 Nebraska Center for Virology, University of Nebraska Lincoln, Lincoln, NE 68583, USA; 3 Botany and Microbiology Department, Faculty of Science, Benha University, Benha 13518, Egypt; 4 Department of Biological Sciences, Faculty of Science, King Abdulaziz University, Jeddah 21589, Saudi Arabia; 5 Department of Microbiology, Immunology and Veterinary Public Health, College of Veterinary Medicine and Agriculture, Addis Ababa University, Bishoftu P.O. Box 34, Ethiopia

**Keywords:** antibiofilm, bacteriophages, biofilm, essential oils, honey, surfactants

## Abstract

Microbial pathogens and their virulence factors like biofilms are one of the major factors which influence the disease process and its outcomes. Biofilms are a complex microbial network that is produced by bacteria on any devices and/or biotic surfaces to escape harsh environmental conditions and antimicrobial effects. Due to the natural protective nature of biofilms and the associated multidrug resistance issues, researchers evaluated several natural anti-biofilm agents, including bacteriophages and their derivatives, honey, plant extracts, and surfactants for better destruction of biofilm and planktonic cells. This review discusses some of these natural agents that are being put into practice to prevent biofilm formation. In addition, we highlight bacterial biofilm formation and the mechanism of resistance to antibiotics.

## 1. Introduction

In 1971, Marshall et al. were the first to develop the concept of bacterial biofilms that was later described by Characklis, Fletcher, and Costerton as “ a unique growth pattern and strong association among microbes that provide specific assets in their life cycle” [1,2,3,4,5]. A more comprehensive explanation of biofilm was provided by Flemming and Wuertz, who defined biofilm as groups of microbes with a distinct multicellular life cycle that undergo division and form clusters, microcolonies, and larger groups [5]. A biofilm can be formed on every surface of higher eukaryotes, including humans [6]. The best example of biofilms that occur in eukaryotic habitats are biofilms that exist as dental plaques, and on skin and guts. In addition, biofilms are formed on different medical devices used in a variety of clinical settings, which act as a vehicle for the transmission of infections [7]. The cells found in biofilms resist adverse environmental conditions as they are encapsulated within a protective hard shell called extracellular polymeric substances (EPS) [8,9]. EPS are made up of polysaccharides, lipids, proteins, and extracellular DNA (eDNA) and play a crucial role in the pathogenesis of several bacterial infections [10].

Biofilms are formed and developed in a complex process involving multiple stages. These stages might be the potential sites for natural antibiofilm agents in tackling biofilm formation and enabling the deep penetration of microbes into the biofilm. Due to the rise in antimicrobial resistance associated with biofilms and the inefficacy of conventional antibiotic therapies, researchers have been evaluating several types of natural compounds to prevent and eliminate the formation of biofilms [10,11].

Bacteriophages (phages) are naturally occurring antibiofilm agents that have shown promising inhibitory and protective effects against the biofilms of several pathogenic bacterial strains [12]. Phages could be applied either individually, as a monophage therapy, or in a group known as a phage cocktail against biofilms [12]. Current studies suggested that the antibiofilm effect of phages is not limited to the parent phage rather, it involves enzymes such as endolysin and depolymerase enzymes which are encoded by the genome of the parent phages. In some cases, these enzymes displayed superior potential compared to the individual phage treatment [13,14]. In addition, genetically engineered phages are also designed to encode antibiofilm enzymes that can be released during infection.

Apart from phages, other naturally occurring agents such as honey, plant extracts, essential oils, and biosurfactants are known for their potential effect against biofilms produced by several bacterial pathogens [13,15,16,17]. These antibiofilm agents exert their mechanism through their peptides, proteins, or other substances that can disrupt the structural organization of the biofilm and/or directly affect the viability of the cellular constituents of the biofilm [18]. This review provides an overview of the role of bacteriophages and natural antibiofilm agents in destroying biofilm formation. In addition, it highlights the process of bacterial biofilm formation and the mechanisms of antibiotic resistance that occurred in the biofilm.

## 2. Bacterial Biofilms

Biofilms are an integrated mass of bacterial cells surrounded by EPS which are related to adherence to both abiotic and biotic surfaces [19]. EPS is composed of lipids (1–40%), extracellular nucleic acids (1–10%), extracellular polysaccharides (40–95%), proteins (1–60%), microbial cells (2–5%), and other substances [20]. Approximately 90% of the total biofilm mass is composed of a matrix, while less than 10% is composed of microorganisms [21,22]. Obviously, differences exist in the chemical and physical components of biofilm among different bacterial species depending on the availability of nutrients, stress levels, type of microorganisms, and host environment [23].

The matrix of a biofilm provides structural support and stability and safeguards the microbial communities from physical and chemical hazards. In addition, the extracellular matrix performs alternative functions, including mediation of migration and colonization, providing signal targets, capturing of cations, and facilitating genetic exchange [24]. Channels and pores within the biofilm help in the circulation of water, gases, nutrients, and other essential chemicals within the matrix and among the different biomasses of biofilm and the exterior environment. It has been known that water (97%) is the principal constituent of the matrix which bathes the functional and architectural units of the matrix [19]. In general, biofilm-forming bacteria can resist the inconducive surrounding environment and survive exposure to both physical and chemical treatments.

### 2.1. Bacterial Biofilm Formation

Bacterial biofilms are produced in a multi-step process that involves physical interactions and chemical signaling among cells and within the same cell. This cell-to-cell interaction involves crosstalk among two-component systems (TCS), quorum sensing (QS) systems, and diguanylate cyclase (DGC) [25]. The TCS, composed of response regulators and histidine sensor kinase, controls signal transduction via a secondary messenger, cyclic di-GMP (c-di-GMP), or phosphorylation, which enables the modulation of gene expression at specific DNA binding sites. As part of the TCS system, the c-di-GMP level depends on phosphodiesterase activities and cytoplasmic or membrane DGC. c-di-GMP-based signal transduction involves an allosteric modification of enzymes, riboswitches, transcription factor interactions, and alteration in post-translational and transcriptional pathways within the cell. These c-di-GMP activities lead to a shift of bacteria from a free-living to a biofilm state [25].

**Figure 1:**
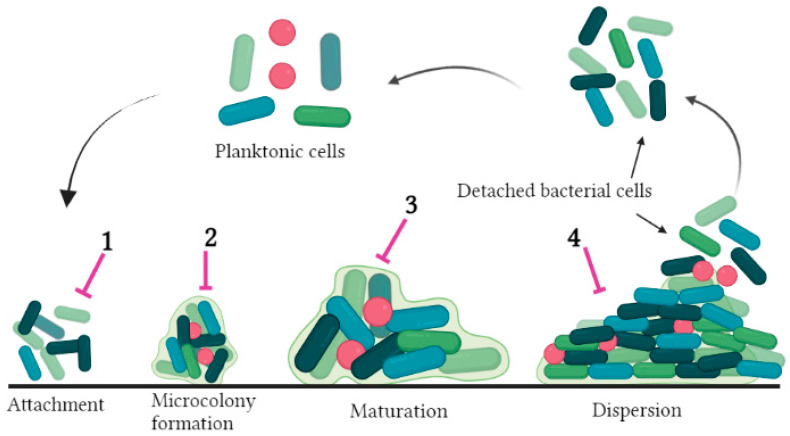
Schematic diagram of the stepwise process of biofilm formation and antibiofilm strategies. Possible antibiofilm strategies: (1) Surface modification and antimicrobial coating; (2) Modulating cell adhesion genes and inhibiting matrix (EPS) formation; (3) Preventing expression of efflux pump; (4) Inhibition of quorum sensing.

The QS state comprises two principal components, the receptor and the autoinducer (AI). AIs are small signaling diffusible molecules synthesized by bacteria that are sensed by receptors once they reach a certain concentration [26]. Gram-positive and Gram-negative bacteria produce oligopeptides (AIP) and N-acyl homoserine lactones (AHL) as signaling autoinducer molecules, respectively [26,27]. By attaching to their receptors, AIs activate specific genes, such as those responsible for antibiotic resistance and biofilm formation [25]. Biofilms produced by bacteria within the same species or upon the interaction of different species or inter-kingdom signal among fungi, plants, and host cells, indicating that bacterial cells in a biofilm interact by crosstalk, self-communication, and receiving signals from each other [28]. Biofilm production proceeds in four steps: (1) attachment (adhesion), (2) microcolony formation, (3) maturation, and (4) dispersion (Figure 1).

#### 2.1.1. Adhesion

Bacterial adhesion to biotic or abiotic surfaces is the initial step of biofilm formation. The process of adhesion can be either reversible or irreversible [26]. Reversible attachment is a temporary adhesion of the planktonic bacterial cell onto a substratum with weak nonspecific bonds such as electrostatic, van der Waal’s, or Lewis’s acid-based electronic forces [29]. On the other hand, irreversible attachment is a permanent adhesion, which results in the firm adhesion of bacteria onto a substratum, facilitated by flagella and pili (fimbriae). Bacterial attachment is influenced by the nature of the substratum, EPS composition, substratum hydrophobicity, and flagella–fimbriae coordination. In comparison with smooth surfaces, a higher adhesion is generated on rough surfaces. Similarly, due to the reduction in repulsion forces between the surface and bacterial cell, surfaces with hydrophobic properties, such as plastic and Teflon, offer a stronger adhesion than surfaces with polar properties, such as glasses and metals [27,29].

#### 2.1.2. Microcolony Formation

Following the attachment of planktonic cells on the surface of a substratum, bacterial cells multiply and divide to produce three-dimensional (3D) aggregates and clusters called microcolonies. The proliferation of microcolonies results in the development of an EPS matrix, aggregation, and adherence of cells, microcolonies protection, and stabilization of the 3D architecture [30]. The EPS is structurally composed of carbohydrates, proteins, and eDNA which shields the microcolonies from adverse conditions including the host immune response, metallic cations, antimicrobials, oxidation, and mechanical removal with greater drug tolerance.

As a physical barrier, the EPS can trap or sequester essential substances to produce a nutritional gradient for the proper diffusion of metabolites, oxygen, signaling molecules, inorganic ions, and other factors across the 3D structure of a biofilm [31]. Microcolonies usually consist of diverse types of micro-communities that organize with each other for metabolic product diffusion, exchange of substrates, and excretion of metabolic waste products. The EPS of Gram-negative and positive bacteria is polyanionic or neutral and cationic, respectively [27].

#### 2.1.3. Maturation

The microcolonies’ formation from small clusters and layered cells leads to the synthesis of a thin film that initiates the maturation process, with the development of an EPS matrix. The bundles of microcolonies result in macrocolonies with the substratum cells displacement to form voids and channels which support the exchange of waste products and nutrients by infiltrating fluid into the biofilm [31]. The core of the matrix is made from polysaccharides whereas eDNA participates in horizontal gene transfer. The maturation of biofilms via signaling molecules results in conformational changes and changes in the genetic expression coding for several virulence factors. Maturation is characterized by a loss of cellular components which are used for motility by expressing flagella-free phenotypes, decrease in phospholipase C and protease synthesis, reduction in the production and eviction of toxins, and synthesis of rough (occasionally mucus-type) polysaccharides [25].

#### 2.1.4. Dispersion

Dispersion is the last stage of the biofilm formation, which causes the cells in the biofilm to shift into a planktonic growth phase to occupy new surfaces and establish a new set of biofilms. Dispersion is an active event of biofilm formation that is triggered or induced by self-produced signaling molecules like fatty acids, and certain environmental factors such as oxygen depletion, shortage of nutrients (starvation), iron, and nitric oxide, which finally lead to a decrease in c-di-GMP levels through a post-transcriptional modification cascade [32]. Low levels of c-di-GMP cause the upregulation of genes that participated in the motility of the cell, including the synthesis of flagella or chemotaxis, and genes and enzymes involved in EPS matrix degradation, such as endA, an endonuclease enzyme that disintegrates DNA found in the matrix, and glycoside hydrolases such as *pelA* and *pslG* which cleave the polysaccharides of the EPS matrix (i.e., *Psl* and *Pel*). Concomitantly, genes involved in EPS or polysaccharide synthesis and fimbriae synthesis are downregulated [32].

### 2.2. Antibiotic Resistance in Bacterial Biofilms

**Figure 2:**
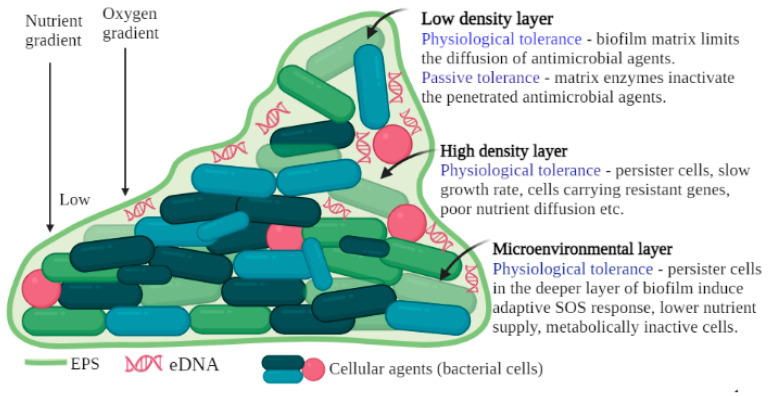
The three principal layers of mature bacterial biofilm (high-density layer, low-density layer, and microenvironmental) and the characteristics of each layers.

The formation of biofilms poses a serious threat to human health throughout the world. Infections caused by bacteria living inside protected biofilm communities are often resistant to antibiotics and such resistance is associated with the structural and functional features of a biofilm (Figure 2). According to the literature, the susceptibility of bacteria in biofilms to antibiotics is about 1000-fold lower than that of planktonic bacteria [33,34].

Infectious diseases associated with biofilms are believed to account for over 80% of chronic diseases, and conventional antibiotics are ineffective in eliminating these infections [35,36,37]. Irrespective of the location of the biofilm, bacteria found on them are resistant or tolerant to the response of the host immune system, antiseptic agents, antibiotic therapy, germicides, and disinfectants [38].

The resistance of bacteria to antimicrobial agents is multifaceted, and it is related to different molecular defense mechanisms used to protect them from the hostile environments in which they exist. A variety of intrinsic and acquired resistance mechanisms play a role in creating difficulties in controlling biofilm formation. It has been reported that microbes in biofilms are resistant to antibiotics via one of the following mechanisms: (i) the contact between antibiotics and the biofilm matrix which adversely affects its activities, (ii) slow bacterial growth rate, (iii) hiding the target sites or genetically modifying the target cells, (iv) the action of modifying enzymes, (v) the formation of persister cells that are tolerant to antibiotics, (v) modulating the chemical microenvironment, (vii) involvement of multiple species of bacteria, and (viii) the biofilm age (Figure 2). Thus, the antimicrobial tolerance and multifactorial characteristics of bacterial biofilms are a serious challenge for the use of conventional antibiofilm therapeutic approaches [38,39].

A bacterial EPS encamps bacterial communities together, which results in a multicellular structure. This multicellular structure is controlled by a phenomenon termed quorum sensing, which allows bacteria to communicate with each other and protect themselves from adverse extrinsic and intrinsic factors, including antimicrobial compounds [40]. As far as *P. aeruginosa* biofilms are involved, Chua et al. reported that colistin-tolerant subpopulations developed during the course of the infection. The cells tolerant to colistin migrated via type IV pili toward microcolonies which became dead due to antibiotic-treated biofilms. These cells initiated the formation of new biofilms via QS [41]. The QS components of *E. fecalis* and other species have been recognized as factors that determine the development of biofilms in the presence of antimicrobial agents [40]. Interference with the QS system increases the susceptibility of *S. aureus* biofilms to different antibiotic classes [42]. Multicellular behaviors such as cell-to-cell signaling, and cellular migration contribute significantly to the formation of biofilms and resistance to antimicrobial agents. A high-density EPS matrix and its ability to bind antimicrobial agents to form an effective barrier that can prevent antibiotics from reaching the various layers of the biofilm [43]. Furthermore, antimicrobial compounds and their toxic derivatives can bind to this matrix and decrease its activity via the enzymatic system or antibiotic chelation [44]. For instance, exopolysaccharides could protect *P. aeruginosa* biofilms from aminoglycosides by directly binding them [45].

Persister cells are a small population of bacteria (0.1–10% of the entire population) that grow slowly or are arrested in their growth. A lack of diffusion of nutrients and oxygen into the periphery of the biofilm contributes to their presence inside the biofilm structure. A fascinating feature of persister cells is their high tolerance to antibiotics. This form of resistance is not associated with genetic factors [46]. These cells can resist 1000-fold the minimum inhibitory concentration of various antibiotics [47]. As a result, persister cells seem to be responsible for the inability of antibiotics to treat chronic infections [48].

Bacterial cells in biofilms encode antibiotic resistance enzymes, such as aminoglycoside adenylyl transferases, which can inactivate or modify antibiotics. Such enzymes are produced and diffused into the matrix of a biofilm to prevent the antibiotics from accessing the nonresistant cells [49]. As an example, a β-lactamase produced by *K. pneumoniae* biofilms effectively degrades ampicillin and prevents it from reaching other susceptible cells [49,50].

Antibiotic tolerance in bacterial biofilms may also be associated with the oxygen gradient across the mass of a biofilm. The antibiotic resistance associated with this gradient is most likely due to the slow metabolic activity of bacterial cells in an anaerobic condition which in turn prevents cellular adhesion and alters the mechanism of action of antibiotics [51]. Moreover, the diffusion of nutrients into the biofilm will affect the density of the cells in the biofilm. Metabolic dormancy has been observed at the bottom surface of bacterial biofilm. Such metabolically inactive cells could be resistant to antibiotics as has been reported in the biofilms of *P. aeruginosa* [52].

Most of the biofilm communities comprised a diverse type of bacterial species. Due to the strong correlation among the various cellular components, the multispecies nature of biofilms may result in greater tolerance to extrinsic factors, including disinfectants and antibiotics. This feature of a biofilm is partly due to the size of the biomass and/or the composition of the EPS matrix [53,54].

The age of the biofilm is one factor that influences the activity and effectiveness of antibiotics. Several communities of biofilms enter the stationary phase with time, indicating that older biofilms display resistance to antibiotics. Chen and coworkers reported that the mature biofilms formed by *S. aureus or P. aeruginosa* were more challenging to eliminate by using conventional antibiotics. This is mainly because of differences in the structure of the biofilm and differences in the composition and/ or function of the EPS matrix [49,55].

### 2.3. Antibiofilm Activities of Phages

**Figure 3:**
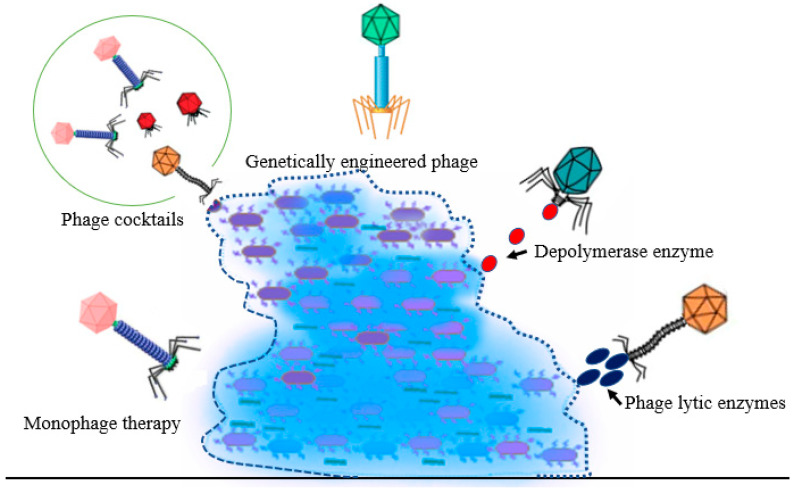
Schematic illustration of the different types of phage preparations (formulations) that can be used against biofilm formation and/or preformed biofilm (i.e., monophage therapy, phage cocktail, genetically engineered phage, depolymerase enzyme and phage lytic enzymes). The blue big mass represents the mature bacterial biofilm.

The resistance of bacterial biofilms to host immunity and antibiotics has led to the search for alternative approaches for the efficient elimination of bacteria in the buildup of the biofilms and antimicrobial-resistant mutants. Phages and their derivatives have been reported as powerful alternative agents in treating and preventing infections associated with biofilm formation (Figure 3). However, to establish successful phage-based treatments, we need a comprehensive understanding of phage resistance and its mechanisms and the evolutionary relationships between these two biological entities. Different phage-based preparations including mono phages, a cocktail of phages, genetically modified phages, and phages encoding enzymes could be used to destroy bacterial biofilms [56,57,58].

#### 2.3.1. Application of Mono Phages

Mono phages which are used on bacterial biofilm should be obligatory lytic and devoid of genes encoding bacterial toxins, virulence, and antimicrobial resistance (AMR). Moreover, the phage genome should not harbor lysogenic genes that mediate horizontal gene transfer. Individual phages commonly have a narrow spectrum of activity as they are limited to a specific strain of the same bacterial species. Their use as biocontrol and therapeutic agents in veterinary, clinical, food, and environmental isolates is immense and inspiring [59]. For instance, phages PSTCR6 and PSTCR4 showed effective reduction of *Providencia stuartii* biofilms developed in catheter models [60].

The human saliva phage, SMHBZ8, showed antibiofilm activity against the biofilm produced by *S. mutans* in a cariogenic dentin model [61]. In the same study, sewage phage isolates inhibited the formation of *S. mutans* biofilm (around 97% of the total mass) by inhibiting the activity and expression of genes which participated in biofilm formation [62]. These two studies indicated that mono-phage preparations could play a vital role in treating dental caries. In a different study, a small concentration of phages pSp-S and pSp-J inhibited biofilm formed by methicillin-resistant *S. pseudintermedius* isolated from veterinary workers and canines [63]. Several mono phages have been utilized to reduce or inhibit viable bacterial cells in biofilms with minimal toxicity to mammalian cells. Research findings have indicated that the concentration of phages is one factor that determines the activity of mono phages. High phage concentrations result in the obliteration of biofilm, while low dose application of phages may not be efficient to penetrate and destroy the biofilm. Hence, the time of exposure of the biofilm to the treatment is the main factor for the disruption of the biofilm in comparison with the concentration of phages [63].

The phenotypic properties of some temperate phages may make them useful in removing biofilms. This phage can be transformed into a lytic phage through genetic engineering. Scientists, for instance, modified the *E. fecalis* lysogenic phage ΦEf11 by removing all genes associated with lysogeny, to reduce the biomass developed by vancomycin-sensitive and resistant *E. fecalis* isolates [64].

#### 2.3.2. Application of Phage Cocktails

A mixture of phages (phage cocktail) is developed by combining two or more lytic phages targeting one or several bacterial pathogens. The rationale behind phage cocktails is to simultaneously target bacterial receptors in diverse antibacterial pathways. This will effectively reduce the bacterial burden, improve host range coverage, enhance lysis potential, and prevent the development of phage-resistant strains [65]. In comparison to mono phage therapy, phage cocktail preparations, as reported in several study models, showed superior efficacy in biofilm elimination [63,66].

A recent study showed that a cocktail comprised of four phages efficiently suppressed the growth of MDR *E. coli* and halted biofilm formation. Nearly 87% of the formed biofilm had been destroyed [67]. To enhance the efficacy and broaden the lytic spectrum of phage cocktails, polysaccharide-degrading enzymes, and the associated phage-encoded enzymes can be included in the preparation. According to a study, a cocktail of four phages lysed all the tested *K. pneumoniae* strains, although one phage lacked depolymerase enzyme encoding genes that are involved in biofilm degradation [68]. Similarly, a cocktail of three phages (ΦKpnM-vB3, ΦKpnP-vB2, and ΦKpnM-vB1) showed significant antibiofilm activity against *K. pneumoniae* biofilm and was lytic towards the tested *E. coil* and *K. pneumoniae* strains [69].

A cocktail of phages is primarily composed of lytic phages, but temperate phages can also be used alone or in combination with lytic phages to develop a cocktail for therapeutic and or biocontrol uses. A cocktail of four temperate phages, Trsa220, Trsa222, Trsa205, and Trsa207, was able to remove 65% of the biofilms formed by *S. aureus* as well as lyse two-thirds of the isolates [70]. Phage cocktails have wide host coverage, making them superior to monophage preparations in reducing the biomass of mixed species biofilms. Mixing phage cocktails AB-PA01 and AB-SA01, which target *P. aeruginosa* and *S. aureus*, respectively, significantly reduced mixed-species biofilm biomass compared with their corresponding individual treatments [71]. A cocktail of a couple of phages, philPLA-C1C, and philPLA-RODI, displayed a decrease in the concentration of adherent bacterial cells to approximately 2 log units in the biofilm of *S*. *epidermidis* and/or *S. aureus* [72]. According to these studies, phage cocktails are superior to individual phages in destroying bacterial biofilms.

#### 2.3.3. Genetically Engineered Phages

Phages that are devoid of genes that encode essential enzymes can be genetically modified to produce biofilm-attacking enzymes for proper attachment and deep penetration of the phages via the EPS matrix, which in turn leads to the destruction of mature biofilms [73]. For instance, a modified *E. coli* phage, T7DspB, was designed to release an intracellular hydrolase during infection. The release of this hydrolase enzyme enhances the degradation of biofilms in the extracellular matrix. The biofilm dispersing (DspB) enzyme, when expressed efficiently following the application of T7DspB on *E. coli* biofilms, degraded almost the whole biofilm (99.997%) and the viable cell count was reduced by 4.5 orders of magnitude, which was approximately 100 times greater than the parent T7 phage [20]. Similarly, an engineered phage, T4 Rnl1, showed higher antibiofilm efficacy instead of lytic activity against *S. mutans* [74]. Currently, some lysogenic phages which lack some lytic enzymes were converted to lytic phages by removing harmful genes and incorporating endolysin-encoded genes which are crucial for biofilm destruction and elimination [75].

#### 2.3.4. Antibiofilm Activities of Phage-Derived Enzymes

Some bacteriophages encoded enzymes in their genome that have shown superior potential against bacterial pathogens and their biofilms. Two major phage degradation enzymes are used to attack bacterial biofilms: depolymerases and lysins.

(i) Lysins

Lysins (endolysins) are murein hydrolases or phage-encoded hydrolytic enzymes which cleave bacterial cell walls during the last stage of the replication cycle. It includes amidase, muramidase, glucosaminidase, transglycosylase, and endopeptidase. Due to a protective outer membrane layer, endolysins are less efficient against Gram-negative bacteria; however, the use of membrane permeabilizers significantly improved their effectiveness [76,77]. Recent genetic engineering techniques have enabled researchers to develop lysin/cationic peptide combinations called Artilysins: bacteriocin–lysin combinations to generate Lysocins, and a combination of lysin and receptor-binding proteins of phages to produce Innolysins [78]. Phage endolysins have been studied for their antibiofilm activities for the past few decades and potential results were obtained. Some of these findings are discussed below and the rest are presented in Table 1. A virulent phage phiIPLA-RODI known to produce an endolysin is called CHAPSH3b [79] and such a combination will lead to a synergistic effect in the eradication of *S. aureus* biofilms, with a significant reduction in the concentration of viable cells in contrast to using either the phage or endolysin alone. Confocal microscopy and time–kill curves indicated that CHAPSH3b decreased the bacterial load up to 7 h following administration, which consequently prevented the regrowth of phage-resistant mutants [79]. The amidase compartment of the vB_LmoS_293 phage showed inhibitory activity against *L. monocytogenes* biofilms on abiotic surfaces. It has been shown that *S. pyogenes* biofilms, which are unresponsive to antibiotics, were efficiently demolished by PlyC endolysin with minimum eradication of concentration compared to standard antibiotics [80]. Lysin CF-301 eliminated *S. aureus* and mixed-species biofilms on food cutting boards, knives, polystyrene, and surgical and catheter surfaces, with a greater antibiofilm effect when pooled with lysostaphin, a cell wall hydrolase [81]. Similarly, endolysin LysCSA13 displayed a significant effect in removing biofilms (80–90%) produced by Staphylococcal strains on several surfaces including polystyrene, stainless steel, and glass [82]. In a different study, endolysin Abtn-4, the endolysin of *A. baumannii* bacteriophage D2, was reported to have wide bactericidal activity against *Enterococcus*, MDR *S. aureus, K. pneumoniae, P. aeruginosa,* and *Salmonella* spp. Abtn-4 had the potential to prevent biofilm formation and possessed lytic activity against phage-resistant strains of the tested pathogens [83].

(ii) Depolymerases

**Figure 4:**
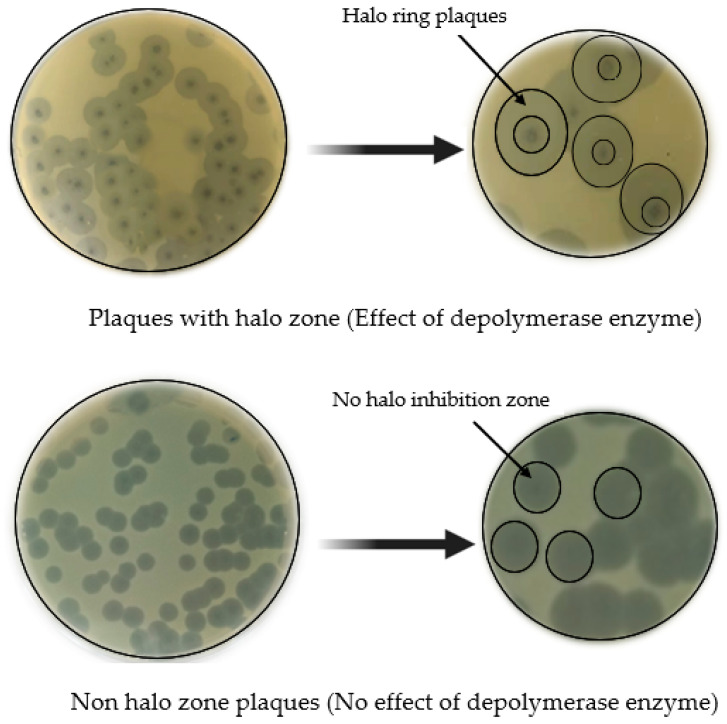
Characteristics of phages with depolymerase and without depolymerase activities in in vitro double agar layer culture plates.

Phage-encoded depolymerase enzymes are proteins that specifically adhere to bacterial EPS compounds and digest them, which disrupts the functional and structural integrity of the biofilm [93,94]. In culture, upon lysis of infected cells, the depolymerase enzyme is emitted as a free enzyme. Under favorable conditions, phages expressing this enzyme will diffuse out on the culture dish (Figure 4) and the size of the zone may increase over time [95]. Several in vitro and in vivo studies indicated that these enzymes showed antibiofilm activity against pathogenic bacterial strains [72,94,96].

In a study conducted by Gutiérrez et al., a depolymerase enzyme, Dpo7, generated from the vB_SepiS-phiIPLA7 phage was evaluated against Staphylococcal biofilms. The results revealed that over 90% of the *Staphylococcus* biofilms were removed by Dpo7 except for the polysaccharide-independent *S. aureus* V329 biofilm. Moreover, the pre-treatment of polystyrene surfaces with Dpo7 resulted in a substantial decline in the biomass of the biofilms (53–85%). This enzyme has the potential to inhibit and disperse *S. aureus* and *S. epidermidis* biofilms [72].

Currently, it has been reported that the TSP depolymerase enzyme of phage ɸAB6 degraded *A. baumannii* biofilm and revealed substantial inhibition of biofilm production and destruction of already-formed biofilms. Furthermore, TSP reduced the inhabitation of the surfaces of catheters by *A. baumannii*, suggesting that it can be utilized to prevent the development of *A. baumannii* biofilms on the surface of medical devices [96]. In a different study, the SH-KP152226 phage-derived recombinant tail fiber protein Dep42 exhibited specific catalytic actions in the depolymerization of the *K. pneumoniae* capsule (K47) and prevented the formation of a biofilm and/or degraded the existing biofilms. The study also indicated that the combination of Dep42 and antibiotics improved polymyxin activity towards *K. pneumoniae* biofilms, suggesting that the cocktail of phage depolymerases and antibiotics may result in significant positive outcomes in treating MDR and biofilm-associated infections [94].

A combination of depolymerase and lysin produced efficient activity in removing biofilms. In a study by Olsen et al. on the efficacy of endolysin LysK and depolymerase DA7 against Staphylococcal biofilms, LysK and DA7 eliminated biofilms from glass and polystyrene surfaces at low concentrations. The combination therapy of these two enzybiotics diminished the concentration of viable cells in comparison to individual enzyme treatments [97]. Some of the depolymerase enzymes that showed antibiofilm activities toward different pathogenic bacterial strains are summarized in Table 2.

### 2.4. Other Natural Antibiofilm Agents

#### 2.4.1. Plant Extracts

**Figure 5:**
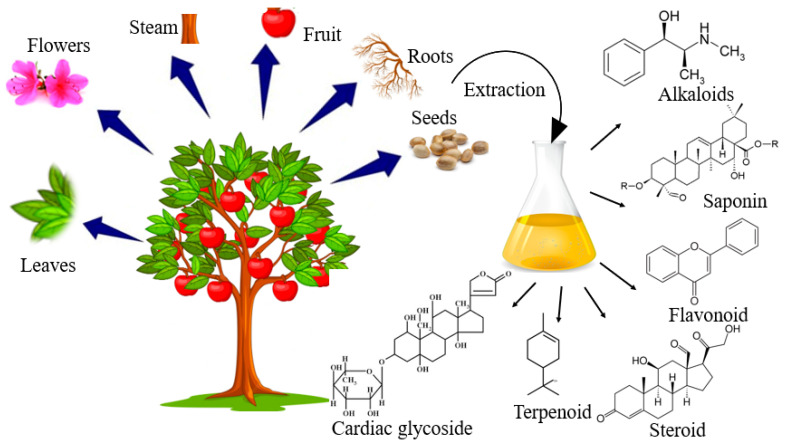
Schematic illustration of the major bioactive compounds (phytochemicals) of plant extracts which have antimicrobial and/or antibiofilm activities.

Plants have been found to contain many compounds that are reported to have antimicrobial properties [106,107], thus becoming an important resource for useful and novel antimicrobial compounds including flavonoids, terpenoids, alkaloids, peptides and polypeptides, tannins, saponins, anthocyanins, quinones, phenolic acids, and simple phenols [108] (Figure 5). It has been recognized that natural products from plants may provide the foundation for discovering new antimicrobial substances with a broad range of bactericidal activities [108]. Accumulating evidence has indicated thatbioactive extracts of several medicinal herbs have the potential to kill pathogenic bacterial strains and destroy the biofilms produced by them [109,110].

A wide variety of plant extracts and their derivatives have been tested for their effectiveness in eradicating bacterial biofilms [109,110]. Some of the recently reported extracts and their antimicrobial and antibiofilm activities are discussed below and summarized in Table 3, which showed that, out of the 119 plant extracts evaluated for antibiofilm activity, five (*Rhodiola crenulata, Malus pumila, Dolichos lablab*, *Polygonum cuspidatum,* and *Epimedium brevicornum*) exhibited strong antibiofilm activities against *E. coli* biofilms. Among these, *E. brevicornum, Polygonum cuspidatum*, and their derivatives (i.e., icartin and resveratrol) showed antibiofilm activity even at concentrations lower than the minimum inhibitory concentration (MIC). The extract of *Melia dubia* was assessed at 30 mg/mL [111]. In addition, these extracts displayed inhibition of swarming motility, hemolysis, hydrophobicity, and *E. coli* biofilm formation. A group of researchers also reported related findings using the extract obtained from *Capparis spinosa* (caper bush). The extract inhibited EPS and biofilm synthesis associated with *Serratia marcescens*, *P. aeruginosa*, *E. coli*, and *P. mirabilis* at 2 mg/mL concentrations [112].

One study found that the fruit extract of the medically significant plant ‘*Lagerstroemia speciosa*’, typically found in Southeast Asia, inhibited the ‘*P. aeruginosa*’ PAO1 biofilm at a concentration of 10 mg/mL [113]. In a different study, fresh garlic (*Allium sativum*) extract (FGE) showed inhibitory activity against *P. aeruginosa* biofilms [114]. A group of researchers also reported the antibiofilm activity of four plant extracts of *Hippophae rhamnoides, Vaccinium oxycoccos*, *Juglans regia*, and *Azadirachta indica*, and among these, *Azadirachta indica* (also called Neem) displayed superior potential in lowering and eliminating *M. smegmatis* biofilms [115].

*Croton nepetaefolius* plant extract (*casbane diterpene*) was studied for its antibiofilm activity and showed inhibitory activity against five Gram-negative pathogenic bacterial species (*P. aeruginosa*, *E. coli, K. pneumoniae, Klebsiella oxytoca,* and *Pseudomonas fluorescens*), two Gram-positive bacterial species (*S. aureus* and *S. epidermidis*), and three yeast species (*Candida tropicalis*, *Candida glabrata,* and *C. albicans*) [115]. In another study, the biofilms formed by *Candida* spp. was significantly reduced by *Boesenbergia pandurata* (“finger root oil”) by nearly 63–98% at 4 to 32 μL/mL MIC levels [116].

In a recent study, the antibiofilm activities of two plant extracts, green tea (*Camellia sinensis*) and Dandasa (*Juglans regia*) were investigated against *Streptococcus mutans* and *E. coli*, and the results indicated that both showed antibiofilm effects at 6.2 and 12.5 mg/mL and 12.5 and 3.1 mg/mL concentrations for *S. mutans* and *E. coli,* respectively [117].

**Table 3:** Recent reports on some of the plant extracts and their antibiofilm activities.

Plants	Extract Type	Target Bacterium	Antibiofilm Effects	MIC	Reference
Piper betle leaf	Ethanol extract	*E. coli* ATCC25922, MRSA, *S. aureus* ATCC25923,	Inhibited biofilm production and promoted its eradication	0.31–2.5 mg/mL	[118]
Dried ground materials of *Camellia japonica* and *Thuja orientalis*	Methanol extracts	*S. mutans* and *C. albicans*	Showed bactericidal activity and inhibited biofilm formation	0.5 mg/mL	[119]
Leaves of the Myrtaceae family	Acetone crude leaf extracts	*Bacillus cereus, E. fecalis, S. aureus*, *E. coli, P. aeruginosa, S.* Typhimurium	Destroyed pre-formed biofilms and halted formation of biofilms	0.04–0.08 mg/mL	[109]
Dried plant material of *Prosopis laevigata*, *Opuntia ficus-indica*, and *Gutierrezia microcephala*	Methanol extracts	Nosocomial microorganisms (*K. pneumoniae*, *E. fecalis*, *E. coli*, *Stenotrophomonas maltophilia*, *S. aureus*)	Showed both antimicrobial and antibiofilm activity against the tested strains	0.7 mg/mL	[120]
Leaf extract of *Pongamia pinnata*	Methanol extract	*Bacillus cereus,* *B. licheniformis*	Showed antibiofilm activity	NA	[121]
Cladodes of Opuntia ficus-indica	Polyphenolic extracts	*S. aureus*	Prevent in vitro and in vivo biofilm formation	2000 µg/mL	[122]
Leaves of *Syncarpia hillii*	Methanol extract	*Staphylococcal* species	Enhanced antibacterial and antibiofilm activities were observed	0.63 mg/mL	[123]
Leaf extracts of *Glochidion lanceolarium*, *Semecarpus anacardium*, and *Bridelia retusa*	Phenolic extracts	*P. aeruginosa, E. coli*, and *S. aureus*	Inhibited biofilm formation	NA	[124]
*Myrtus communis* (Myrtenol)	Ethanol extract	*S. aureus*	It had antibiofilm activity and in silico results indicated a good pharmacokinetic profile	128 µg/mL	[125]
Leaf extracts of *Ocimum gratissimum*, *Alchornea laxiflora, Morinda lucida, Ficus exasperata, Jatropha gossypiifolia,* and *Acalypha wilkesiana*	Acetone, methanol, and ethanol extracts	*S. aureus, E. fecalis, Salmonella* spp., *E. coli, Campylobacter* spp., and fungal species (*Aspergillus fumigatus, Aspergillus flavus*, and *C. albicans*	Showed good antibiofilm activity (>50%) against at least one organism	0.03–0.15 mg/mL	[13]
Buds of *Populus alba* and *Populus nigra* extracts	Methanol, ethanol, and ethyl acetate extracts	*S. aureus*, *E. fecalis, Bacillus subtilis, Listeria innocua, E. coli, P. aeruginosa, C. albicans, Saccharomyces cerevisiae*	Showed antimicrobial and antibiofilm activities against the tested species	NA	[126]

NA—not available.

#### 2.4.2. Honey

Honey is a nutritious natural product prepared from the nectar of melliferous plants by bees [127]. Honey is a common and widely used product that can be used to heal wounds, counter inflammation, fight bacteria, and protect against oxidative damage [127]. The antibiofilm potential of different types of honey has been reported [128,129]. A relatively low concentration of honey can prevent microbes from transferring virulence genes and expressing curling QS, which contributes to biofilm formation. The antimicrobial properties of honey may also prevent bacteria from adhering and forming biofilms. Despite its antibacterial activity, honey also prevents the formation of biofilms with its antibacterial agents such as bee defensin 1 [130]. This and other peptides directly attack the viability of biofilm-producing microbial agents which indirectly halts the formation of biofilms [128]. Different types of honey of Korean and American origins have been shown to downregulate the expression of multiple genes [*ycfR* (*BhsA*), *csgA*, *yifo* (*bsmA*)] involved in biofilm formation [131].

Previous studies on honey demonstrated that it has antibiofilm activity against the biofilms of *K. pneumoniae* and *P. aeruginosa* [132], oral Streptococci [133,134], *Proteus mirabilis* (*P. mirabilis*), and *Enterobacter cloacae* (*E. cloacae*) [134]. Among the monofloral types, manuka honey is one of the most studied, and researchers have demonstrated its ability to inhibit the biofilm formation of *Clostridium difficile* [135], *S. aureus* [136], and *C. albicans* [137]. In a recent study by Lu et al., manuka honey inhibited the formation of *P. aeruginosa* biofilms, halted the growth of the planktonic cells, and eradicated established (pre-formed) biofilms at a concentration of 8–32% [95]. Similarly, Balázs and colleagues investigated the antibiofilm and antibacterial effects of Hungarian locust, black, sunflower, and linden honey towards selected biofilm-forming respiratory tract bacteria (*P. aeruginosa*, *Hemophilus* spp., and *Streptococcus pneumoniae*). The results indicated that all four honey samples suppressed the growth of the tested strains and inhibited the formation of biofilms [138].

Apart from the above-mentioned reports, several other studies evaluated the antibiofilm effects of different types of honey; however, some of these studies did not characterize the chemical composition and the concentration of the principal antibacterial components such as antimicrobial peptides, hydrogen peroxide, phenolics, or methylglyoxal, which vary based on the geographical origin of the honey and its floral base as well [139].

#### 2.4.3. Essential Oils (EOs)

Since ancient times, humans have used essential oils as aromatic extracts and for culinary purposes, as well as in folk medicine, due to their wide range of pharmacological properties including antiseptic, anti-inflammatory, and analgesic properties [140]. Several pathogenic microbial strains have shown susceptibility to certain EOs and their components [140,141]. Unlike Gram-negative bacteria, Gram-positive bacteria lack an outer membrane which makes them more susceptible to EOs. The cell wall and inner membrane of Gram-positive bacteria are hydrophobic, allowing hydrophobic molecules to easily penetrate. The effect of phenolic compounds varies with concentration. At low concentrations, the compounds interfere with enzymes that produce energy, while at high concentrations the compounds denature proteins [142,143].

**Figure 6:**
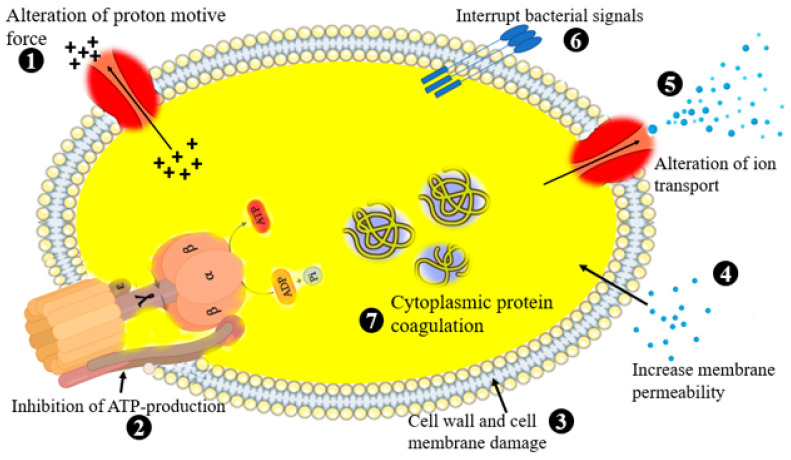
Schematic illustration of the mechanisms of antimicrobial activities of EOs which indirectly influences biofilm formation. Black plus stands for proton.

EOs are composed of a wide variety of volatile biomolecules [144], with many of them containing over 300 different compounds. The broad spectrum activity of EOs is associated with the chemical reactions of alcohols, esters, ethers, amides, amines, terpenes, heterocycles, aldehydes, and phenolic compounds synthesized from secondary metabolism in different parts of the plants [145]. EOs work by interacting with the cell membrane and, as a result, disrupt the integrity of the microbial cell, which leads to cell death [143]. The bioactive components of EOs may have several cellular targets including inhibition of ATP production, cell wall and cell membrane damage, cytoplasmic coagulation, and alteration of ion transport [146] (Figure 6).

EOs are naturally occurring volatile substances derived from plants. These natural products are effective and preferred by the food industry because of their antibacterial and preservative properties. A wide variety of pathogenic microorganisms have been successfully treated with these essential oils since ancient times. EOs have antimicrobial properties, which can destroy microbes by attacking their cell walls. Furthermore, these oils appear to be effective at inactivating many microbes without causing resistance to antimicrobials [147]. Its low toxicity to mammalian cells, quick environmental decomposition, and the availability of diversified essential oils make them potential antibiofilm agents [148,149,150]. Some of the recently reported essential oils and their antibiofilm activities against different pathogenic microbial strains are discussed below and summarized in Table 4.

*Cuminum cyminum* also known as cumin oil is one of the EOs which is derived from aromatic medicinal plants of the “Apiaceae” family and has an astringent effect on the digestive system. In addition to its use as a carminative and eupeptic, it has also been used as an analgesic for acute gastric diseases. In a study performed on *K. pneumoniae* strains, cumin seeds showed decreased biofilm activity while improving ciprofloxacin efficiency [151].

Cinnamon oil is an example of EOs which is produced from the bark of the cinnamon tree “*Cinnamomum zeylanicum*” and cinnamon bush “*Cinnamomum cassia*” and is used in the food industry for its distinctive fragrance. Studies have indicated that this oil can inhibit S. *mutans*, *Lactobacillus plantarum*, *S. epidermidis,* and *S. mutans* biofilms [152].

According to a study, oregano essential oil has antimicrobial properties against *S. hemolyticus*, *S. aureus*, *S. sciuri*, *E. coli*, and *S. lugdunensis* and could prevent biofilm formation. Moreover, it also attacks mature biofilm formation even at exceptionally low MICs. A Brazilian nut oil named *Bertholletia excelsa* (a vegetable oil) was also tested for its ability to inhibit dental biofilm formation on commercially available dentifrice. By adding this vegetable oil to commercially available dentifrice, scientists were able to inhibit the formation of dental biofilms and control periodontal disease [153].

Research groups assessed the synergistic effect of *Melaleuca alternifolia* (tea tree oil (TTO) essential oils and ciprofloxacin against *P*. *aeruginosa* biofilms. According to the results, the combined effect of ciprofloxacin and TTO resulted in a significant reduction in biomass of the biofilms by more than 70% and reduced the number of cells even at the lowest concentration of ciprofloxacin (1.25 g/mL) [154]. In a different study, the effects of thymol and oregano oil, extracted from cinnamon (*Cinnamomum verum*), were investigated for their effects on the formation of biofilms in three biofilm-forming bacterial strains, *Acinetobacter, staenotrophomonas*, and Sphingomonas. It was shown that two out of the three strains were resistant to biofilm formation at MICs. Additionally, among the tested oils, “thyme oil” was more efficient and displayed inhibitory effects even at sub-lethal concentrations of 0.001% (*w/v*) [153].

**Table 4:** Recent reports on the antibiofilm activity of different types of essential oils.

Source of Essential Oils	Active Components	Antibiofilm Activities	MIC	Reference
Clove	Eugenol	*L. monocytogenes* and *S. Enteritidis* biofilms were reduced by 30.2% and 20.3%, respectively.	NA	[155]
Lemongrass (*Cymbopogon flexuosus*)	Citral	Bactericidal against *S. aureus* and *Candida* spp. and the biomass of their biofilms was reduced following treatment.	NA	[156]
*Cymbopogon nardus* and Geraniol	NA	*S. aureus* biofilm biomass was reduced up to 100% at 0.5–4 mg/mL concentrations. Number of viable cells was reduced at 0.25 and 1 mg/mL concentrations of EOCN and geraniol, respectively.	0.5 and 0.25 mg/mL	[157]
*Lippia origanoides*	Thymol, carvacrol, phellandrene	Showed antibiofilm activity against the biofilms produced by *E. coli* O157:H7 and methicillin-resistant *Staphylococcus aureus* (MRSA).	0.4–1.6 mg/mL	[158]
*Lippia alba*	Carvona, citral	*E. coli* O157:H7 and methicillin-resistant *Staphylococcus aureus* (MRSA).	>3 mg/mL	[158]
*Satureja Montana*	Carvacrol	*P. aeruginosa, Streptococcus pyogenes, S. mutans, Streptococcus sanguis, Streptococcus salivarius,* and *E. feacalis Lactobacillus acidophilus*.	15.28 µg/mL–125.00 ± 8.66 µg/mL	[159]
*Cinnamomum zeylanicum*	Eugenol	Antibiofilm activity against the biofilms produced by *Acinetobacter, K. pneumoniae, P. vulgaris, E. fecalis, S. aureus,* and *S. epidermidis.*	0.5–8 mg/mL	[160]
*Rosmarinus officinalis*	1,8-cineole, α-pinene, borneol, camphor, βmyrcene	Showed antibiofilm activity towards *S. epidermidis* S61 and *S. aureus* ATCC 9144 biofilms.	0.312–0.625 μL and 1.25–2.5 mL^−1^, respectively	[161]
*Elletaria cardamomum*	1,8-cineole, linalool acetate, α-terpinyl acetate, sabinene,	Different concentrations of this essential oil prevented biofilm formation by *E. coli* O157:H7 and *S*. Typhimurium JSG 1748 at different rates.	1%	[150]
*Cinnamon* (*Cinnamomum verum*) bark	Caryophyllene, β-thujene, 3-allyl-6-methoxyphenol, acetic acid cinnamyl ester, o-cymene, and α-phellandrene	Antibiofilm activity against maturation of oral biofilms (multi-species).	NA	[161]
Thyme plant	Thymol	4096 and 2048 μg/mL concentration of this oil effectively inactivated an *E. fecalis* population in mature *fecal* biofilms by 7.20 and 5.75 log CFU/mL, respectively, at 30 min post-treatment.	128 and 256 μg/mL	[39]
*Clove essential oil* (*CEO*) and *oregano essential oil* (*OEO*)	Eugenol, eugenol acetate, beta-caryophyllene alpha-humulene	Showed antibiofilm activity against *Salmonella Derby* biofilms.	1/8 MIC	[162]
*Laurelia sempervirens* (*Chilean laurel)*	Safrol, methyl eugenol	Showed high antibiofilm activity against *S. aureus* biofilms at a concentration of 128 µgmL^−1^.	64 µg mL^−1^	[163]

NA—not available; ATCC—American Type Culture Collection, MIC—minimum inhibitory concentration.

#### 2.4.4. Biosurfactants

**Figure 7:**
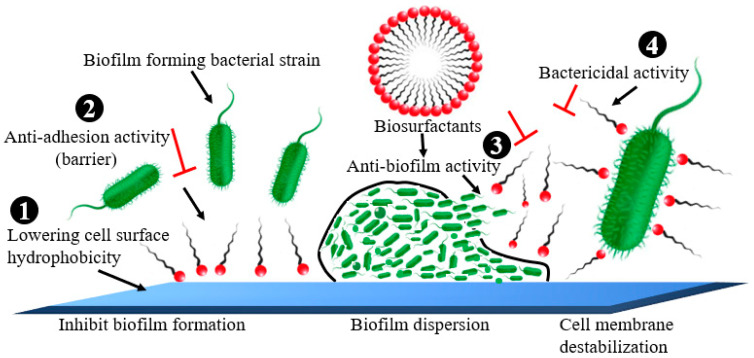
Schematic demonstration of the mechanisms of antimicrobial activities of biosurfactants. (1) Inhibition of biofilm formation by lowering cell surface hydrophobicity or anti-adhesion activity; (2) Inhibition of biofilm formation by act as a barrier between cells and the contact surface—anti-adhesion activity; (3) Biofilm dispersion (antibiofilm activity against the preformed biofilm); (4) Cell membrane destabilization (bactericidal activity).

Biosurfactants (BS) are microbial amphiphilic compounds which are either secreted outside or act on the surface of the host cell [164]. It is used by pesticide, cosmetic, biodegradation, agriculture, oil, food, and pharmaceutical companies. BS displayed strong anti-adhesive and antimicrobial characteristics, making them effective against pathogenic bacterial strains and their biofilms [164]. BS prevented the formation of biofilms by altering the cell adhesion effect via lowering cell surface hydrophobicity, inhibiting the electron transport chain, and membrane disruption thus limiting the demand for cellular energy [165] (Figure 7).

Different types of biosurfactants are produced by several microorganisms that showed antifungal, antibacterial, and antibiofilm actions [166]. For instance, the activity of biosurfactants generated from *Pediococcus acidilactici* and *Lactobacillus plantarum* on QS signaling molecules and expression of biofilm-associated genes in *S. aureus* were assessed [167] and the results indicated that biosurfactants reduced the formation of *S. aureus* by regulating biofilm-associated gene (*icaA*, *dltB*, *cidA*) expression. *Lactobacillus plantarum* BS lowered expression of the *cidA* gene at 12.5 mg/mL [167]. *Pediococcus acidilactici-*derived BS reduced the gene expression of accessory gene regulator (*agr A*), autoinducer-2 (AI-2) signaling molecules, and staphylococcal accessory regulatory (*sar A*) at 50 mg/mL [168]. It has previously been shown that Lactobacillus-derived BS-loaded liposomes exhibit greater antibiofilm activity than free BS, inhibiting MRSA biofilm formation and lowering the severity of the infection [169]. For more information, the details about the antibiofilm activity of different types of BS have been previously reviewed in [170].

#### 2.4.5. Maggot (Fly Larval) Therapy

Maggot (fly larval) therapy with the larvae of *Lucilia sericata* is an effective and simple method for cleaning infected and necrotic wounds [171,172]. The use of this therapy dates back to the beginning of civilization, and it became globally prevalent and popular for the treatment of infected or chronic wounds during the 1930s [173]. In the 1940s with the introduction of antibiotics, however, the interest in this natural surgery unfortunately disappeared. However, in the 1990s, the emergency of antibiotic resistance resulted in the revival of maggot therapy. To date, maggot therapy, despite its repeated fall from favor as well as the continued public disdain that hampers its acceptance, is on the rise because of its effectiveness, simplicity, and safety [174].

Maggot excretions and secretions (ES) have been investigated both for their potential to inhibit bacteria from forming biofilms and to disrupt pre-formed biofilms. A preliminary investigation into the effects of maggot ES on biofilms found that different types of bacteria responded differently to maggot ES in terms of bacterial biofilms [175].

Freeze-dried ES inhibited the formation of *S aureus* biofilms, while maggot ES induced the formation of *P. aeruginosa* biofilms until 10 h following application. ES were able to degrade *S. aureus* pre-formed biofilm, whilst over a 10-fold concentration was required to degrade *P. aeruginosa* pre-formed biofilms [175]. Such species-based differences were also observed in a subsequent investigation. Maggot ES significantly reduced the formation of biofilms by *E. cloacae* and *S. aureus*, while the development and growth of *P. mirabilis* were not affected [176]. These findings indicated that maggot ES may act specifically against different strains, instead of showing a broad spectrum of activities.

It has been also observed that maggot ES inhibited the development of biofilm formation associated with two *Staphylococcus epidermidis* strains (1457 and 5179-r1) that displayed different mechanisms of action, thus suggesting that there may be more than one biomolecule or mechanism of action involved in the antibiofilm or antibacterial activities of maggot ES [177].

In a separate investigation, maggot ES showed a preventive role in the formation of biofilms and disrupt the pre-formed *P. aeruginosa* biofilms on the surfaces of medical devices. According to the report, these results could be associated with the accumulation of ES which was generated from third-cycle maggots. In addition, the authors also observed the antibiofilm activity of ES against *S. epidermidis* and *S. aureus* [178]. Additionally, dried *L. sericata* larvae-derived fatty acids were found to have antibiofilm formation activity against *Streptococcus pneumonia* and *S. aureus* [179]. Moreover, a purified DNase extracted from maggot ES was found to degrade extracellular bacterial DNA in *Pseudomonas* biofilms. Bacteria need extracellular bacterial DNA (their own DNA or other bacterial DNA) to build up a biofilm; therefore, the maggot DNase capable of degrading all extracellular DNA suppressed the process of biofilm formation [180].

Studies on biofilms have shown that, although maggot ES can degrade and disintegrate biofilms of different species, the bacteria released by these biofilms are not destroyed [176]. This was investigated further by Van der Plas et al. who indicated that biofilms resisted antibiotics alone, but the combination of antibiotics and maggot secretion (daptomycin, vancomycin, or clindamycin) caused the destruction of *S. aureus* biofilms and the eradication of the bacteria found in the biofilms [181]. This introduces a strategic approach to the treatment of biofilm-associated infections, whereby the use of a combination of maggot ES and antibiotics could generate more successful therapeutic outcomes than the use of either one of these methods alone [182]. For instance, a study conducted by Arora and colleagues indicated that the combined use of maggot ES with ciprofloxacin displayed higher antimicrobial activity in comparison to the individual application [183]. Two other antibiotics, flucloxacillin and gentamicin, also showed higher synergistic antibacterial activity with maggot ES than either one of these individually [184].

## 3. Conclusions

Due to the emergence of biofilm-producing, antimicrobial-resistant pathogens, alternative (natural) antibiofilm agents are urgently needed and have been sought to tackle biofilm-associated infections more than ever before. As shown above, phage and phage-derived antibiofilm agents have shown promising efficacy towards biofilms of different bacterial species regardless of some limitations. In addition, other naturally occurring antibiofilm agents including honey, plant extracts, essential oils, and surfactants have shown promising antibiofilm activities against the biofilms produced by different bacterial pathogens. However, in some cases, the antibiofilm efficacy needs to be enhanced by emulsifying or combined each other or with other related antibiofilm agents for better clearance of the biofilm. Hence, further research is needed in combination therapy and much work is needed to discover new natural antibiofilm agents.

## Figures and Tables

**Table 1:** List of phage endolysins and their source and antibiofilm activities.

Phage (Endolysin)	Host Bacterium	Antibiofilm Activity	Reference
PA26 (LysPA26)	*P. aeruginosa*	Reduced the cells in the *P. aeruginosa* biofilm by 1- to 2-log CFU and destroyed the biofilm matrix.	[84]
phi68 (Lys68)	*Salmonella*	Reduced biofilms when coupled with malic or citric acid.	[85]
C1(PlyC)	*Streptococcus pyogenes*	Degraded biofilm matrix.	[80]
SMP (LysSMP)	*Streptococcus suis*	Efficient towards 32 biofilm-forming strains and >80% destruction of biofilms resulted	[86]
CSA13 (LysCSA13)	*Staphylococcus*	Destroyed the *Staphylococcus* biofilms on glass, stainless steel, and polystyrene surfaces. The mass of the biofilms was reduced by about 80–90%.	[82]
GRCS (PlyGRCS)	*Staphylococcus*	Active against planktonic and biofilm forms of MRSA.	[87]
Phi SAP-2 and 11 (LysSAP-2 and LysPhi11)	*Staphylococcus*	Eliminated whole biofilms created on polystyrene surfaces.	[88,89]
Phi84 (Lys84)	*S. aureus*	Approximately 90% of the biofilms of *S. aureus* were destroyed.	[90]
ClyR (LysClyR)	*S. sobrinus* and *S. mutans*	Reduced the viable cell counts in 72 h aged *S. sobrinus* and *S. mutans* biofilms following treatment at a concentration of 50 µg/mL, for 5 min.	[91]
ECD7 (LysECD7)	*E. coli*	Showed antibiofilm activity towards a wide range of bacterial biofilms including biofilms of *K. pneumoniae* Ts 141-14 clinical isolate.	[92]

**Table 2:** List of depolymerase enzymes encoded by different phages and their antibiofilm activities.

Phages	Family	Depolymerase Enzyme	Targeted Species for Antibiofilm Activity	Reference
vB_EcoM_ECOO78	*Myoviridae*	Dpo42	*E. coli* (Clinical isolate)	[98]
PHB19 (T7-like phage)	NA	Dep6	Shiga toxin-producing *E. coli* (STEC)	[55]
Petty	*Podoviridae*	Dpo1	*A. nosocomialis* and *A. baumannii*	[99]
IME180	*Zobellviridae*	NA	*P. aeruginosa*	[100]
Phage P560	*Podoviridae*	P560dep	KL47 type *K. pneumoniae*	[101]
vB_PmiS_PM-CJR	*Siphoviridae*	NA	*P. mirabilis* BB2000	[102]
ΦK64-1	*Myoviridae*	Multiple depolymerases	*Klebsiella* capsular types	[103]
KP34	*Podoviridae*	KP34p57	*K. pneumoniae*	[104]
NA	NA	*Aeromonas punctata*-derived depolymerase	*K.* *pneumoniae*	[105]
ISTD and NOVI	*Myoviridae*	NA	ISTD resulted in a 2- and 3.5-log decline in planktonic and viable bacterial cells in the biofilm and planktonic cells, respectively.	[56]

NA—not available.

## Data Availability

Not applicable.
